# Supplementary material for: Mechanisms and Effect of Coptidis Rhizoma on Obesity-Induced Inflammation: In Silico and In Vivo Approaches
Source: Int J Mol Sci. 2021 Jul 28;22(15):8075. doi: 10.3390/ijms22158075 (PMC8347796; doi:10.3390/ijms22158075)
Supplement: Supplementary file 1 [file ijms-22-08075-s001.zip › ijms-1298516-supplementary.pdf]

Supplementary Materials

Mechanisms and Effect of Coptidis Rhizoma on Obesity-Induced Inflammation: *in silico* and *in vivo* Approaches

Oh-Jun Kwon, Ji-Won Noh and Byung-Cheol Lee*

Department of Clinical Korean Medicine, Graduate School, Kyung Hee University, 26 Kyungheedae-ro, Dongdaemun-gu, Seoul 02447, Korea

* Correspondence: hydrolee@khu.ac.kr Tel.: +82-2-958-9151; Fax:+82-2-958-9182

Supplementary Figures

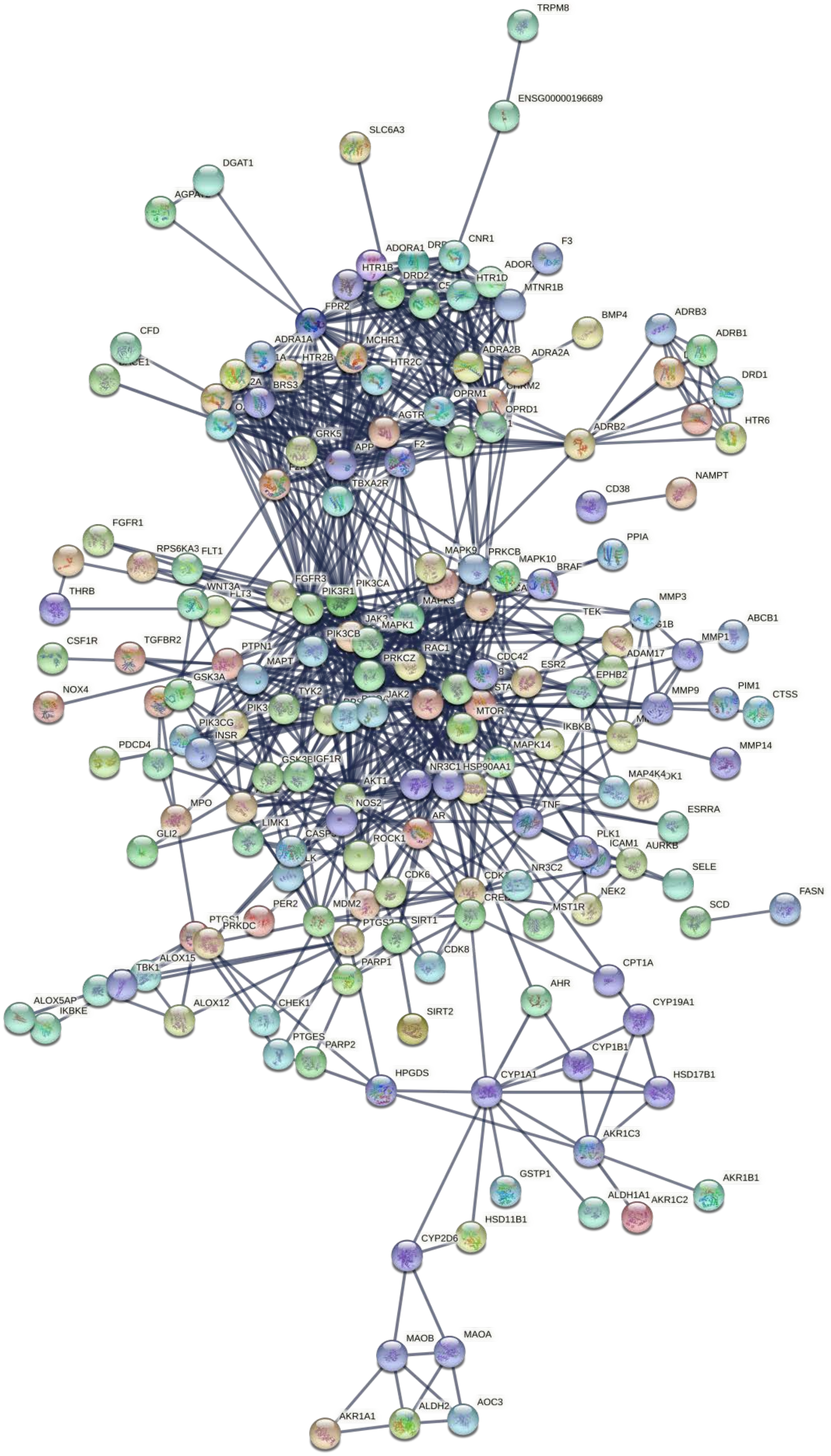

Supplementary Figure S1. Protein-protein interaction of 217 overlapping genes

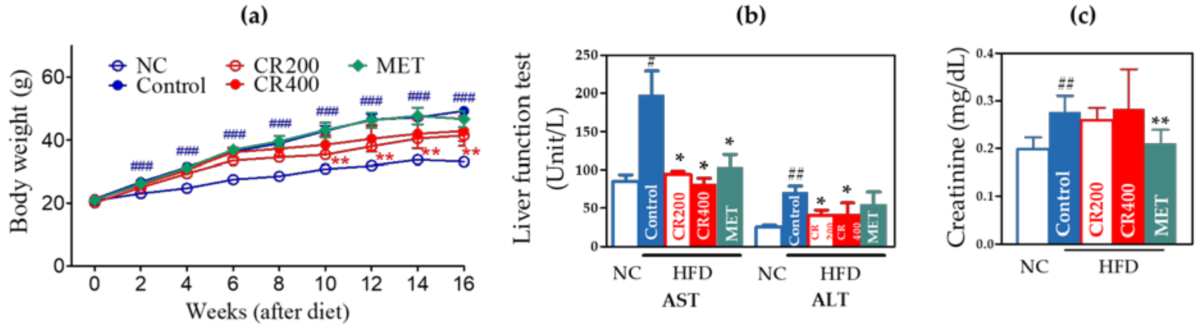

Supplementary Figure S2. Body weight, liver and kidney function test. (**a**) Body weight changes of experimental period, (b) AST and ALT levels, (c) Creatinine levels. Data are expressed as mean ± standard error of the mean (SEM). # p < 0.05, ## p < 0.01, ### p < 0.001, control compared with NC, and * p < 0.05, ** p < 0.01, *** p < 0.001, CR 200, CR 400 and MET compared with control. NC, normal chow; HFD, high-fat diet; MET, metformin.

Supplementary Tables

Supplementary Table S1. 12 bioactive ingredients of Coptidis Rhizoma obtained from TCMSP

Mol ID	Molecule Name	OB (%)	Caco-2 permeability	DL
MOL001454	**Berberine**	36.86	1.24	0.78
MOL013352	**Obacunone**	43.29	0.01	0.77
MOL002894	**Berberrubine**	35.74	1.07	0.73
MOL002897	**Epiberberine**	43.09	1.17	0.78
MOL002903	**(R)-Canadine**	55.37	1.04	0.77
MOL002904	**Berlambine**	36.68	0.97	0.82
MOL000622	**Magnograndiolide**	63.71	0.02	0.19
MOL000785	**Palmatine**	64.6	1.33	0.65
MOL000098	**Quercetin**	46.43	0.05	0.28
MOL001458	**Coptisine**	30.67	1.21	0.86
MOL002668	**Worenine**	45.83	1.22	0.87
MOL008647	**Moupinamide**	86.71	0.55	0.26

Supplementary Table S2. Target genes of 12 ingredients of Coptidis Rhizoma obtained from SwissTargetPrediction

	**Target genes of 12 ingredients of Coptidis Rhizoma**
**Berberine**	HTR2B BCHE ADRA2C ADRA2B CHRM1 ACHE SIGMAR1 CYP2D6 SAE1 UBA2 CDC42 RPS6KB1 AURKA DHFR RAC1 AURKB CYP11B2 GRIA1 HTR3A GABRB3 GABRA3 GABRG2 GABRB3 GABRG2 GABRA1 GABRB3 GABRG2 GABRA5 IKBKB CHEK2 MAP2K1 HPGD PPIA SLC1A3 TBXAS1 MAPKAPK2 PRF1 PARP2 PIK3CD PIK3CB PIK3CG ABL1 CHRM4 KIT LCK SRC CHEK1 TYMS PTGS2 JAK2 CDK2 CDK4 BCAT2 EPHB4 TRPC6 ICAM1 SELE ALOX5AP MAOB MAPK14 ROCK2 LIMK1 SCN4A PIM1 PFKFB3 F3 PARP10 HSD17B1 MET CSF1R RPS27 MAPK8 HCRTR2 EPHX2 ZAP70 LYN TEK TRPM8 PTPN1 HSD17B2 CCNA2 CDK2 CYP11B1 FLT3 NTRK1 HSD11B1 MKNK1 CDC25B
**Obacunone**	OPRK1 MIF OPRM1 OPRD1 MAPK1 CTSS CTSV CTSL P2RX3 BACE2 BACE1 CTSK CASP3 CASP7 HMGCR DRD2 HTR2A PLA2G2A HSD11B1 MDM2 PIM1 CNR2 AOC3 PIM2 CDK5R1 CDK5 DYRK1A FBP1 CFD WNT3A CSF1R PDE7A HTR7 PARP2 TNKS2 PSEN2 PSENEN NCSTN APH1A PSEN1 APH1B CCR1 MAPK14 SRC KDR MAPK11 PTGES PREP FAP AVPR1A C5AR1 MAOB NAMPT SMYD2 F2R BRS3 LRRK2 SCN5A SCN9A CENPE OXTR CYP51A1 CNR1 MAPK8 EPHX2 CHRM3 MAP2K1 PER2 FASN P2RX7 EPHX1 CHRM2 CHRM1 SIGMAR1 AKR1C3 CCNE1 CDK2 GABRB3 GABRA3 GABRG2 GABRB3 GABRG2 GABRA1 GABRB3 GABRG2 GABRA5 GABRA2 GABRB3 GABRG2 CDK1 PARP1 NR1I2 AURKA CYP19A1 RET PDE2A PDE10A LDHA TAAR1 HSP90AA1 ACHE SORD ITK PDE9A CDK2 CCNA1 CCNA2 PFKFB3 GRM5 RHOA CLK4 CLK1 CLK2 DYRK1B LIMK2 HTR2B
**Berberrubine**	HTR2B BCHE ADRA2C ADRA2B CHRM1 ACHE SIGMAR1 CYP2D6 SAE1 UBA2 CDC42 RPS6KB1 AURKA DHFR RAC1 AURKB CYP11B2 GRIA1 HTR3A GABRB3 GABRA3 GABRG2 GABRB3 GABRG2 GABRA1 GABRB3 GABRG2 GABRA5 IKBKB CHEK2 MAP2K1 HPGD PPIA SLC1A3 TBXAS1 MAPKAPK2 PRF1 PARP2 PIK3CD PIK3CB PIK3CG ABL1 CHRM4 KIT LCK SRC CHEK1 TYMS PTGS2 JAK2 CDK2 CDK4 BCAT2 EPHB4 TRPC6 ICAM1 SELE ALOX5AP MAOB MAPK14 ROCK2 LIMK1 SCN4A PIM1 PFKFB3 F3 PARP10 HSD17B1 MET CSF1R RPS27 MAPK8 HCRTR2 EPHX2 ZAP70 LYN TEK TRPM8 PTPN1 HSD17B2 CCNA2 CDK2 CYP11B1 FLT3 NTRK1 HSD11B1 MKNK1 CDC25B
**Epiberberine**	SAE1 UBA2 ACHE HTR2B BCHE ADRA2C ADRA2B CHRM1 SIGMAR1 CYP2D6 CDC42 RAC1 TBXAS1 TGM2 RPS6KB1 AURKA DHFR SCD HPGD CHEK2 HTR3A ICAM1 SELE CYP19A1 NTRK1 KIT LCK SRC CD38 TYMS NR3C2 MAOB PARP1 PIK3CG F3 ALOX5AP JAK2 CYP11B2 IMPDH2

	AURKB GRIA1 MAPKAPK2 MAPK10 ROCK1 PTPN1 PRKACA XBP1 ADORA2A ADORA3 PIK3CD PIK3CB TRPM8 SIRT2 PIM1 PIM2 ABL1 RPS27 IKBKB MAPK8 PTGS2 AGPAT2 SIRT3 SIRT1 GRK5 FLT1 BCAT2 MAP4K4 EPHA2 RPS6KA3 MST1R ZAP70 MET AXL EPHX2 NQO2 MKNK1 DRD4 DRD3 CCNC CDK8 GRM1 PRF1 CDK8 FGFR1 STAT3
**(R)-Canadine**	DRD1 DRD2 F3 HTR7 HTR1A CHRM4 ADRA1D DRD3 SIGMAR1 DRD4 ADRA1A SLC6A3 ADRA1B DRD5 HTR2A HCRTR1 ADRB2 ADRB3 HTR2C ADRB1 TBXA2R ABCB1 HTR2B MAOA MTNR1B ADRA2C BCHE ACHE DHCR7 PIK3CA PIK3R1 CDC7 CHRNB4 CHRNA2 CHRNA3 CHRNB2 KCNN3 GRIA1 PIK3CD PIK3CB PIK3CA HCRTR2 CHRNA4 CHRNB2 ADRA2B CDK2 CCNA1 CCNA2 KCNN1 KCNN2 MTNR1A MKNK1 PPP1CA CTSL SCN9A CDK5R1 CDK5 CYP19A1 CCNB3 CDK1 CCNB1 CCNB2 MAPK8 CYP11B1 MAPKAPK2 CYP11B2 PARP1 PTGS2 SLC1A3 TGFBR1 MAOB ESR1 HTR6 ADRA2A ABCC1 CDK9 CHRNA3 CHRNB4 ABL1 GCK RPS6KB1 AURKA BTK OPRM1 NR3C2 ADORA1 PDE10A HTR1B NTRK1 CHEK1 SLC18A2 CTSK PLK1 GSTP1 GSTM2 PLK3 GABRB3 GABRA3 GABRG2 GABRB3 GABRG2 GABRA5 GABRA2 GABRB3 GABRG2 CDK2 CDK4 QTRT1 NEK1 TYMS AURKB CSNK1G1 CHEK2 RPS6KA1 MET ATR
**Berlambine**	HTR2B BCHE ADRA2C ADRA2B CHRM1 ACHE SIGMAR1 CYP2D6 SAE1 UBA2 MKNK1 DRD2 F3 F2 CDK2 CCNA1 CCNA2 DYRK1A FLT3 PIK3CA TGFBR1 RAC1 CLK4 CDC7 CCND1 CDK4 MAPK8 MAPK9 GAK GABRB3 GABRA3 GABRG2 GABRB3 GABRG2 GABRA1 GABRB3 GABRG2 GABRA5 GABRA2 GABRB3 GABRG2 PIM1 PFKFB3 SLC1A3 CLK1 CLK3 DYRK2 CDK2 CDK4 SCN9A TTK DHFR ABL1 EIF2AK3 HSP90AA1 MAPKAPK2 CBFB PIK3CA PIK3R1 MAPK10 ROCK2 PGK1 ITK MET CDC42 NAAA CDK9 CCNT1 MAP2K1 RPS6KB1 ALOX5 PIK3CD PIK3CG MAPK3 ALDH3A1 MAPK1 AURKA PTGES MAOB AURKB RPS6KA3 BACE2 LCK CSNK1D IRAK4 BACE1 PLK3 TRHR ILK PIK3CB PRF1 HTR3A KIT CA7 CTSK GSK3A PLK1 CA14 CPT1A CSF1R ALDH1A1 ABCB1 AXL CHEK2 RPS6KA1 JAK2 GRM5 ALOX5AP ATP4B ATP4A TRPC6 ROCK1 PRKACA P2RX7
**Magnograndiolide**	PTGS2 CYP19A1 POLB HSD11B1 BRD4 BRD2 FKBP1A AR CDC7 PRKCA F2 PRSS1 CTRC TERT DRD2 JAK1 JAK3 TYK2 JAK2 LRRK2 NOS2 PTGES CYP11B1 CYP11B2 NR3C1 CDK5R1 CDK5 PTGS1 KCNA5 TRPM8 PDE10A ABCB1 TAAR1 MPO CREBBP PRKDC PARP1 LIPA HTR1D ABCC9 KCNJ11 ABCC9 MAPK14 ADRB2 ADRB1 ADRB3 PIK3CD PIK3CB CYP51A1 AOC3 CXCR2 PDCD4 TLR8 TLR7 POLA1
**Palmatine**	ACHE HTR2B BCHE ADRA2C ADRA2B CHRM1 SIGMAR1 CYP2D6 RAC1 SAE1 UBA2 CDC42 AURKA MAP2K1 AURKB TEK CDK2 CCNA1 CCNA2 PLK1 PLK3 PLK2 EPHB4 LCK MET LYN CFD CSF1R DYRK1A MAPK8 MAPK10 ROCK2 PRF1 HTR3A MAPKAPK2 PGK1 MCHR1 MAPK9

	HCRTR2 PIK3CD PIK3CB PIK3CG NAAA CYP1B1 ALDH2 TTK LTA4H ABCG2 ADORA3 CLK4 CDC25B RAF1 BCAT2 MST1R BMP4 CYP1A1 CCNC CDK8 MDM2 CDK8 TGM2 GRM5 GRM1 GCK MTOR PTGS1 NTRK1 CTSS CDK2 HSP90AB1 CHEK2 PRKCB CDK4 MKNK2 TGFBR2 SIRT2 MKNK1 PDK1 ATR PPIA DHFR KIT IKBKE KDM5B TRPV1 TBK1 SCD FLT3 FGFR3 HSD17B2 HSD17B1 PIM1 ZAP70 CLK1 DYRK2 KARS MARK1 PDGFRB EPHX2 FPR2 PIM3 FLT4 JAK3 JAK1
**Quercetin**	NOX4 AVPR2 AKR1B1 XDH MAOA IGF1R FLT3 CYP19A1 EGFR F2 CA2 PIM1 ALOX5 AURKB DRD4 ADORA1 CA7 GLO1 MPO PIK3R1 ADORA2A DAPK1 PYGL CA1 GSK3B SRC PTK2 HSD17B2 KDR MMP13 MMP3 CA3 ALOX15 ABCC1 PLK1 CA6 CDK1 MMP9 CA12 MMP2 PKN1 CA14 CA9 CSNK2A1 ALOX12 MET CA4 NEK2 CXCR1 CAMK2B ALK AKT1 ABCB1 NEK6 PLA2G1B CA5A BACE1 CYP1B1 AXL ABCG2 NUAK1 AKR1C2 AKR1C1 AKR1C3 AKR1C4 CA13 AKR1A1 GPR35 SYK MAPT KDM4E TOP2A INSR ACHE MYLK PIK3CG APEX1 CDK5R1 CDK5 CCNB3 CDK1 CCNB1 CCNB2 ARG1 PTPRS ESR2 MPG SLC22A12 CDK6 CDK2 TYR HSD17B1 AHR ESRRA APP PARP1 TTR MMP12 CD38 AKR1B10 TNKS2 TNKS TERT
**Coptisine**	HTR2B BCHE ADRA2C ADRA2B CHRM1 ACHE SIGMAR1 CYP2D6 SAE1 UBA2 CDC42 RAC1 DHFR CHEK2 HPGD CHRM4
**Worenine**	ACHE SIGMAR1 CHRM1 SAE1 UBA2 HTR2B BCHE ADRA2C ADRA2B CYP2D6 CA14 CA2 XBP1 RAC1 CHRM4 PRKCE PRF1 ALOX5AP AURKA DHFR LIMK1 FLT3
**Moupinamide**	MMP9 MMP2 MMP1 EGFR CNR2 MAOB TYR BRAF CDK1 PTGS2 ALOX5 SYK CHEK1 WEE1 CTSL MTNR1A HSP90AA1 BCHE ACHE GLI2 ABL1 CDK5R1 CDK5 ALDH2 MTOR PDK1 DNM1 ESRRA ESRRB THRA THRB PRKCZ RPS6KB1 CHEK2 BMP1 MMP3 DUSP3 MMP8 CA7 CA6 CA12 CA14 CA9 CA5A ROCK2 HPGDS DRD2 DRD3 HSD17B2 HSD17B1 ADORA1 ADORA2A PDE4D PDE4C HDAC1 CDK1 CCNB1 CCNE1 CDK2 CCNE1 CDK3 ADAM17 GRK2 SLC5A1 MMP13 MMP7 TRPM8 ALK INSR CFD TNF TOP1 AGTR1 ANPEP MMP14 PNMT CDK4 PTPN1 TRAP1 HSP90B1 HTR3A HSP90AB1 VCP AVPR1A NR1H4 ADORA3 ADAM10 TSPO CA13 CA5B TYMS MIF PDE7A EPHA2 MMP16 AKT2 EPHB2 MAP2K1 EPHA5 EPHA4 EPHA8 MMP12 EPHB3

Supplementary Table S3. 2861 obesity-related target genes obtained from Genecards, DisGeNET and OMIM

**Obesity-related target genes**
CELA2A NR0B2 SDC3 LEPR KIDINS220 ADCY3 POMC GHRL PPARG CEP19 CARTPT PCSK1 ADRB2 MRAP2 ENPP1 LEP ADRB3 NTRK2 INPP5E FFAR4 TUB UCP2 UCP3 FTO AGRP MC4R DYRK1B MC3R INS ADIPOQ RETN IL6 TNF CRP SIM1 IGF1 GNAS LIPE LEPQTL1 SERPINE1 GCG UCP1 SH2B1 NPY LPL HSD11B1 BDNF INSR PYY IRS1 CNR1 NAMPT BBIP1 APOB SHBG PPARA APOE RBP4 SLC2A4 GNB3 PRMT7 ACE PPARGC1A CCK TCF7L2 CCL2 GH1 BRCA2 APOA1 MTTP PTPN1 SLC6A14 MKKS BBS4 GHR PNPLA3 SCAPER IRS2 ALMS1 PRL GPT INSIG2 IGFBP1 RARRES2 POLG LIPC BBS1 BBS2 CYP19A1 GCK GLDC NOS3 MIR122 HCRT NIPA1 TMEM18 CDIPT FABP2 ESR1 NR3C1 GNPDA2 MIR143 PHIP TTC8 ADIPOR1 ALB VPS13B IGFBP3 FABP4 IGF2 PLIN1 HDAC8 BBS10 FASN PON1 PPARD PNLIP IL18 ITLN1 IL1B FGF21 AKT1 FNDC5 DRD2 KCNJ11 CETP NUDC HNF1A FAAH PNPLA2 ATP6V0D1-DT MKS1 DPP4 APOA5 GGT1 ADIPOR2 MTHFR SNRPN HNF4A GHRH IL10 AHDC1 IAPP LMNA PHF6 MAGEL2 NEGR1 SERPINA12 GHSR STAT3 ABCC8 SOCS3 SCD C3 SREBF1 SIRT1 GLP1R APOC3 BBS5 DLK1 RIC3 CDKAL1 AOMS2 BBS7 TP53 BBS12 ICAM1 AOMS1 CRH LEPROT COMT HTR2C CAPN10 BGLAP COQ4 KCTD15 TLR4 AGT ADRB1 APLN VEGFA LAS1L SDCCAG8 CLOCK MCHR1 RPS6KA3 SLC6A4 MIR33A SLC30A8 LCN2 EDN1 LPA GAL AHSG CXCL8 PIK3CA BBS9 NR1H2 VDR CD36 SST MFN2 LOC101929710 IGF1R NDN HTR2A MTOR SLC17A5 PRKAB1 ARL6 GNB1 F2 NPY2R GCKR ATAD3A MMP9 AKT2 DGAT1 GAD2 APOA4 IGFBP2 MTCH2 SELE LOC106728418 MKRN3 NPY5R MIR21 ABCA1 LZTFL1 H6PD GRIN2B TGFB1 PTEN KMT2B SLC2A1 WDPCP SLC6A3 PRKAR1A ATP2B3 MAP1B RUBCN NAGLU REN ACACB GIP IRAK1BP1 CPE PRKAA2 NT5DC1 C8orf37 ANGPTL4 EIF2S3 TNFRSF1B NPPB DOCK3 AQP7 ARL14EP NPAP1 MLXIPL ACP1 BDNF-AS LDLR ACACA IL6R EBF3 CPT1A CIDEA LIPE-AS1 PRKAA1 EXOSC3 PDX1 IGF2BP2 CCL5 MTNR1B CCKAR AGER NMB FGFR1 NTS CEP290 BMIQ7 ENPP2 SNORD116@ CYP2E1 HNF1B CLCN6 FKBP14 KARS1 MIR599 LOC110120801 ADRA2A TYR NR1H4 TNFRSF11B PIK3R1 ATP10A NLRP3 SOD2 SLC2A2 PPARGC1B VCAM1 MTM1 TLR2 OXT HSD11B2 TRH HP DRD4 AFF4 MIR126 SLC6A2 CREBBP CTNNB1 TRIM32 PPY IFT27 SIRT3 LBP PMCH ACSL1 FOXO3 GPX3 HK2 CD40 HADH GLUL NPY1R TF NEIL1 NPC1 AHR MYT1L KCNMA1 RAI1 HOXB5 PACS1 DPYD RMST PRKCH TBC1D1 BCHE HSPA5 PTGS2 SAT1 NR1I3 CTSS CYP1B1 HMOX1 MIR34A STS SOD1 TRPV1 NR1I2 CD163 CD68 CES1 KSR2 NHLH2 CTF1 OGG1 PCK1 RAPGEF3 GPX1 ITGAM ACHE SLC22A1 VLDLR CEBPA ADH1B PARP1 CS DDIT3 ZNF169 FCGR3B FTL IDO1 MIR130B MRC1 PFKFB3 PRKAR2B TFRC CA3 AKR1C3 ALDH1L1 LACTB COX7C CPB2 APCDD1 PPM1L CYB5A NQO1 ECHS1 EFNB1 EP300 ESR2 RAB21 ZFR2 GFPT1 HMGB2 ACADM IFNG MIR130A MIR184 ME1 ALDH6A1 MMUT ZFHX3 ACLY NUCB2 ZBTB7B DCXR ACP5 CYCS FGGY CYP26B1 ATPAF1 SLC22A3 SLC22A2 UQCRC2 AKAP1 CASP1 GAS7 PEX11A SUCLG2 SUCLA2 WNT3A SLC16A7 ENTPD6 SIRT6 SERPINF1 STK11 RPS6KB1 NMU GOT2 PRKCB CDKN2A CST3 AR ANGPTL6 CAV1 NR1H3 HRH3 ANKRD26 NNT GUCY2C JAK2 ASIP PRLH BCL2 SNAP25 TSC1 AOC3 SOCS1 ALOX12 GAPDH GLRX HK1 SLC27A1 FADS1 NOS1 PON2 PRKG1 SREBF2 XDH CRHBP DCN DRD1 GABRA6 PRLHR ATP5F1B KDM3A PROX1 BAD PTPRF CCL4 SDC1 TYK2 GALP NCOA1 CDKN1B ADARB1 CTSC EHD1 CPS1 DEFB1 FGG G6PD GIPR GJA5 PCSK1N GPR12 GSTT1 HMGCS1 HTR1B RHOA MAPT KITLG MYC MYOD1 MYOG NCF2 NDUFB6 ATP4B OTC OXCT1 ATP5F1D PDE3A PRKCD PRKCI PLSCR3 GPAM RSC1A1 ABCG5 SFTPB SFTPA1 SLC9A3 SLC10A1 AACS BNIP3 SRD5A1 STAT4 C5AR1 EHMT1 CAV2 FADD FAIM2 DMD BEST1 SEC16B ETV5 APPL1 TFAP2B EGF ARG1 PRDM16 NRXN3 ATP6AP2 DUSP6 CADM2 MAP2K5 RAP1A KISS1R ABCA4 SETD2 HLA-DRB1 SOX6 MTMR9 TBX1

UBE3A HDAC4 CEL LINGO2 EMD ANOS1 MECP2 NF2 PAX6 WWOX WNT4 AHI1 PROK2 TULP1 KMT2D CUL4B ELN ARL13B FGFR3 FTSJ1 GATA4 NEUROD1 PAK3 TNNI3K RAB23 RPTOR RFC2 BLK SOX2 SOX3 WT1 HESX1 ARNT2 MED12 NR2E3 ZGLP1 GHRLOS KCNJ18 BCDIN3D-AS1 FPGT-TNNI3K HUWE1 LINC01524 SCN1A-AS1 TOPORS APC2 MERTK DEAF1 LRRC53 PRPF8 FARS2 LINC02465 LINC02055 PNPLA6 MID2 CRYZL2P-SEC16B IL1RAPL1 PNKP ADCY9 RAB39B CLCN4 CANT1 CNGB1 CNGA1 PROKR2 ZNF513 POC5 HGSNAT PTCHD1 CRX SOCS2-AS1 RDH12 CTSH KLB CYP7A1 DGKG DCC LRRN4 ARX DLG3 DNMT3A AGTR2 MEGF8 ERBB2 ACSL4 FAT1 JMJD1C FGF8 FHL1 CNKSR2 ZNF365 SNRNP200 FOXO1 IQSEC2 POGZ FLII SYNE2 RPGRIP1L TTC28 SYNE1 ARHGEF18 ADNP CRB1 ARL2BP PDSS1 FLRT3 PRPF6 GABPA GABRA3 GABRD FSCN2 SIN3A NSMF COPD PRPF31 IFT172 MYCBP TBL2 GDI1 HS6ST3 RNU1-1 RBMX AMY1A AMY1B AMY1C GNAT2 GP1BB TTC28-AS1 CCDC141 GRN GTF2I GUCA1B SH3KBP1 HCFC1 HIF1A HLA-DQB1 HOXB3 IDH3A IDH3B EYS ZNF81 APP APRT KLK3 IL1A IL17A IMPDH1 ACADVL ITPR3 KIF7 CERKL LHFPL3 KCNH2 PCARE FEZF1 USP27X LIMK1 EML6 LINC01122 CASC15 ARL3 HOXB-AS3 SMAD4 MAK ARVCF MFAP1 NR3C2 MOG MYH9 NCAM2 RERE NEK2 NFE2L2 NINJ1 NPHP1 NRL OTX2 P2RY11 PAX4 PAX5 IMPG2 PCDH9 NIN PDE4D PDE6A PDE6G PDGFB PDE6B SUFU PIK3CB PIK3CD PIK3CG PLG POU3F4 UGT1A10 UGT1A8 UGT1A7 UGT1A6 UGT1A5 ATRX UGT1A9 UGT1A4 UGT1A1 UGT1A3 IL17RD CNNM2 POMGNT1 CHD7 WDR11 ERMARD SPATA7 KIZ ANGPTL8 MAPK1 KLHL7 MAPK8 MRPS22 PTH HACE1 KIAA1549 SLC7A14 SUGP1 HAMP PTX3 RAP1B RBP3 PRPH2 RGR RHO RLBP1 AGBL5 ROM1 RP9 RP1 RP2 RPGR RPE65 RREB1 SAG SCN1A CXorf56 SEMA4A NSD1 SHOX UPF3B RSRC2 SLC5A2 SLC8A1 SMARCB1 SOX10 SPP1 BRAF SRY BRS3 SYP TACR3 TAF1 TBCE TBX3 TCOF1 TSPAN7 HIRA TNFSF4 UFD1 USH2A CLRN1 KDM6A CLIP2 ZNF711 ZNF41 CA4 PRCD CACNA1S CCDC28B TMEM43 IRX3 STEAP4 ZNF408 ALG13 RABEP2 DHDDS GLRA3 IFT74 SPG11 ALPK1 IFT88 SPRY4 USP9X FAM161A CCDC77 KLF11 SLC9A7 OFD1 KCNAB2 SHANK3 FGF17 PROM1 BAZ1B AIP USP8 TMEM67 PRPF4 PRPF3 CDHR1 LRAT REEP6 NRXN1 HS6ST1 ARHGEF6 GDF15 GTF2IRD1 SEC24C IFT140 FRMPD4 DHX38 TRIM66 FGF19 ZBTB7C MSTN ARSA MPO NNMT BAAT SORT1 CIDEC SPARC SPX IL33 CMKLR1 GPBAR1 GRK2 EDNRA LPIN1 GCGR APOA2 MIR155 MAOA PHB CHDH AZGP1 ACKR3 SFRP5 SI MGAM CD59 CXCR6 GPR151 CPT2 NRG4 ADRA1A ADRA2B AGTR1 EPO FBN1 SIRT2 PCSK9 AMH GPR42 IL15 ENHO LGALS3 MIR146A MME MMP2 LGR6 MBOAT4 TNMD SSTR4 THBS1 TNFRSF1A CAT LPAR2 KHDRBS1 ADAMTS13 NISCH MGLL C1QTNF5 MRGPRX3 MRGPRX4 MAPK14 CXADR OXER1 DIO2 EGFR F5 GPRC6A ANGPTL2 LPAR3 MRGPRX1 IL37 ANPEP GOLGA6A ITGAX LECT2 MIR221 MIR27A VN1R17P GPR166P PDK4 AVP RCBTB1 CHPT1 TRIB3 CCND1 BRD2 ATXN2 SGK1 WNT5A CNBP CAD FGF23 FZD4 MBD2 GRAP2 TRIM13 AHSA1 PLIN2 ADM GPR119 CRK F2RL1 F3 FKBP5 NUP62 RNF19A POLDIP2 ANGPT1 GTF2H1 HGF NR4A1 HSPA1A HSPA1B KRT16 LCT ARNTL ARR3 MGAT1 MIF MSRA MTRR MYD88 OPRM1 OXTR DCTN4 BFAR PGR CCHCR1 APOM MAPK3 NMUR2 MOK ACE2 BCL2A1 RENBP RORC ROS1 SAA1 CEACAM1 BMP4 SLC5A1 CXADRP1 SPG7 TLR3 TM7SF2 WNT1 WNT10B XBP1 AIMP2 MFRP SERPINA6 CCN4 SQSTM1 SELENBP1 XPR1 IL32 CD14 GPR55 CDH13 FST METAP2 LOC110806262 CHI3L1 CNTF LYPLAL1 MAP3K8 COX8A CTLA4 DBP DECR1 APLNR EPHB2 ESRRA FGF1 FOXC2 FN1 ANKK1 GC SPINK4 HPGDS ANGPTL3 RABGEF1 FFAR1 GPS2 TBK1 CXCL2 A1CF HMGB1 HSPA4 HSPD1 IFNA1 IFNA13 IL2 INPPL1 KCNQ1 KNG1 ARG2 KRAS C1QTNF12 MCL1 MEST MGAT2 COX2 NFKB1 NHS NM NPPC NOX4 IL22 SCLY PKD1 TERF2IP MAPK9 PSMD9 MIR146B CXCL5 SFTPD CRTC3 TFAM TMPRSS2 TPD52 TWIST1 TRPM8 CALCR SESN2 SYVN1 DGAT2 CREG1 PTER ABCG2 CD38 CD44 FOXO6 LINC01672 PTPRU LRPPRC CDK4 GDF11 LOC102723407 PERCC1 SORBS1 POSTN EBP MTCO2P12 CHGA FSTL1 C1QTNF6 CISH LEAP2 ACVR1C CX3CR1 CYBA CYBB DDT DNMT1 NR0B1 EIF4EBP1 ESRRG ALDH1A1 MMRN1 AKR1B1 ZFPM2 FOS BACE1 ALOX5 FSHR NR5A1 CERS6 NOCT HSPA12A SPAG8 GAS6 PDCD4 ANGPT2 FFAR2 REM1 KLF15 PYCARD GSTM1 GUCA2B RMC1 PSAT1 CFH HOXA5 HTR2B HCAR2 APOC1 LCE1C FAS IL1R1 IL4 IL6ST CXCL10 INSIG1 INSRR IRF5 IRF6 KCNK3 GFRAL C1QL3 LIPA LOX LRP5 MIR148A

MIR199A1 MIR199A2 MIR223 MIR26B SMAD3 MBL2 MMP1 MMP3 ATF4 NDUFAB1 CCN3 OLR1 PRKN TAS2R13 PC SOST PDE3B ARID4B ABCB1 PLA2G1B MEG3 APOBR CTNNBL1 LRRC8A TAS2R38 MIR181D ARRDC3 UBL5 SLC25A19 RNASEL S100A9 SCN10A NOD2 SELENOP SFRP4 HIF3A SLC6A8 GGTLC5P BRCA1 STAT5A STAT5B SULT2A1 ADAM17 BTF3P11 TH THY1 TLE3 TLR5 TSHR CAPN5 TST TTN GGTLC3 GGT2 CCR2 GGTLC4P VPS51 VWF TFEB CALCA HDAC11 NR4A3 MOGAT2 CALR NRIP1 ARID1A MCHR2 FAM71F1 OGT APOL1 TSLP LGR5 HDAC3 PER2 CD1D CD5L MSC KL IKBKE LPIN2 RAPGEF5 BMS1 IP6K1 MED13 ADA C20orf181 CD24 BCL2L11 MIR1275 AAA1 DNM1L BED TOB1 SORBS3 CDK6 PRG4 AP3S2 CDKN1A CBSL CDKN2B HMG20A CEACAM5 LARP1BP2 RNU6-392P CCL27 HPSE NPFFR2 GPR83 MTMR11 SLC27A2 ADCY5 NUDT3 CHIT1 PRRT2 CHRM2 CHRNA2 CHRNA5 ADCY8 UCN3 CHRNB4 C1QTNF1 THEM4 ANTXR2 CLU CCR5 COL6A3 RBM45 COMP COPA COX4I1 CP CPT1B CRABP1 CREB1 CREM FUNDC1 CRY1 HT CSF1R IL34 CCN2 CTSK CCDC80 CUX1 CYP1A1 CYP1A2 CYP2A6 LINC00689 CYP2B6 CYP2B7P CBLL2 CYP27A1 DAG1 DBI SGMS2 ACAN ATN1 RCAN1 TSC22D3 DSPP DUSP1 EGR1 AIF1 ELANE CRTC2 ENPEP PRSS55 EPAS1 ERG ERN1 FBL F13A1 ALDH2 PTK2B FCGR2B FDXR GPC4 FGB FGF2 FGF5 FGF10 FGF13 FGL1 FKBP4 ATF6 NT5C2 KDM4B FOXM1 ZNF423 FLT1 RRS1 FMO3 NEDD4L CRTC1 PADI4 SMUG1 DAPK2 PPP1R15A PLA2G15 CADM1 ALOX5AP PANX1 ZNF318 G6PC BRWD3 GABRA2 BAMBI ASPM PTPN22 AMD1 GEM GFAP GFER HAVCR1 GJA1 GJA4 ACAD8 GK GLB1 SGSM3 GLO1 GNRHR GPD1 CXCR3 HCCAT5 METRNL LPAR4 GPER1 RGMB SLC27A6 SCG3 CD274 GSTP1 REPIN1 NPC1L1 ANXA1 H2AX ANXA2 HFE HHEX HOXD13 HSF1 HSPA2 HSPG2 TNC C1QTNF9 IDE IFNB1 APOD IGFALS IKBKB IL13 ING2 ISL1 EIF6 JAK3 GSTK1 MALAT1 KISS1 KRT7 KRT18 RTL1 LCAT LINC01194 MIR107 MIR10B MIR141 MIR152 MIR15B MIR200B MIR222 MIR23A MIR29A MIR32 MIR96 SMAD2 MAF MAS1 MC1R MC5R DNAJB9 MDK MFAP2 ASCL1 MMP14 EIF2AK4 MIR342 ASPA PLF RNR1 MUSK ATF3 NFATC3 NFE2L1 NGF NNAT NOS2 NPPA MIR425 ATP5F1A ORM1 PAEP REG3A SERPINA5 ABHD5 HSPA14 DNAJC27 TNFRSF12A IL23A KLF13 LUC7L3 PECAM1 PER1 PFKP PGF SERPINA1 PIN1 PKM PLA2G4A PLAG1 PLAT TM6SF2 PLTP PTOV1 PON3 APPL2 QRSL1 LGR4 PRCP PREP SLC30A10 PRKCA SELENOS ACSS2 MYDGF MAPK10 MAP2K3 PROC PROS1 METTL3 CCL28 TMPRSS15 SUCNR1 ATP10D ABHD6 MIR519D ARHGAP21 CFAP97 PTPN2 IL21 RAPSN GER SENP2 RGS2 BDKRB2 S100A8 S100B SAA2 ACSM3 BGN CCL11 CCL19 CCL20 THADA SELP SELPLG XYLT2 CARD9 SETMAR SFRP1 HHIP PYY3 SHC1 CERK P2RY12 IL25 BMP2 PINK1 NCF1 SLC15A1 BMPR1A SLN SNRNP70 SOD3 SORL1 MAP3K7 TAL1 TAZ TBX15 MIR532 TCF21 TEK TERF1 THBD THRB THRSP TIMP1 TIMP3 C1QBP TPH1 CRISP2 C3AR1 TRPC1 TRPM2 TNFRSF4 PLIN4 TYRP1 UCN UROD USF1 VIM VIP WFS1 CXCR4 VKORC1 METRN PRRC2A PLA2G7 MUL1 DHX40 ADM2 WDR26 FLAD1 AAAS TLR10 TAM CAST CASP3 ROPN1L PLA2G6 SGIP1 ATRN IL1F10 SPZ1 ACCS DGKE TNFSF11 KLF7 TNKS TNFSF14 TNFSF10 CBS SOCS2 CCN5 KAT2B SPHK1 ARTN CCT CLDN10 SLC39A13 NLRP12 NOG CD8A KLF4 SLIT2 PPIG SLC22A8 LIPG ATG5 MAGED1 LITAF TBPL1 NPEPPS GDF3 ABCG1 RGS6 KEAP1 DNAJC6 TBC1D4 MVP CDH2 AKT3 MIR675 TANK ZSCAN30 VTRNA2-1 MIR374B MUPP HDAC6 DIP ENDO1 IBD20 INSL5 WAGRO BOP MIR1908 TMED7-TICAM2 CHAF1A PARP2 GJC1 LY75-CD302 TMX2-CTNND1 DEL11P13 KLRC4-KLRK1 COMMD3-BMI1 ABCC5 MIR4777 MIR4460 MIR4443 MIR4517 BIRC6-AS1 CDH11 PPIF PGR-AS1 OPN1MW3 LSINCT5 BCAP31 FAM13A TRIM28 WASF2 DHRS9 RBM6 MACORIS RNF41 CDK5 CALCRL ANGPTL7 TRIB1 PLIN3 RCAN2 CDK8 SPRY1 IRX5 RAMP2 RAMP3 LOC102724197 LOC102724971 RTN3 AKR1A1 KLF2 CITED2 CEPT1 PEMT PIAS3 YAP1 ERICD IFI30 OLFM1 CDX2 TOMM40 MAD2L2 TIMM44 SLC9A6 NCOA2 SEMA4D CEBPB SEMA3C CEBPD KAT5 HYOU1 ZNRD2 THRA1/BTR LINC00237 PRDX4 ARL6IP5 AGPAT2 SLC35A1 OLFM4 DPYSL4 LRP1-AS TXNRD2 SCGN RGS14 CAMKK2 CST12P RN7SL263P CELF1 SIRT1-AS CGRRF1 DCTN6 GNA13 TNFSF13B CETN1 POLD3 ORI6 OGA NFAT5 NES CFTR SEPTIN9 CCR9 WASF3 PPP1R13L LILRB1 ADAM28 USP19 CEACAM8 MRPS30 UTS2 PAPOLA PRDX3 FERMT2 SDS SLC27A5 SLC27A4 RIPK3 CHD2 RAPGEF4 STIN2-VNTR ADAMTS5 PRDM4 CHEK1 WDHD1 MAP4K5 IRAK3 AKAP13 KLHL2 VSIG4 PDAP1 TUSC2 OIP5 CHRNA4 DBA2

TXNRD3 SORCS1 OSBPL11 C1QTNF3 C1QTNF7 HAUS1 H3P23 H3P10 SLC5A11 ADCYAP1 SLC22A12 ABHD15 LRG1 AGAP2 FTSJ3 RLN3 TWIST2 CLCN3 MSS51 CLCN7 GSTO2 CLK2 CLN3 CLTA TPH2 CMA1 SPPL3 CCR6 CCR7 ACKR2 IQCK ZNF543 CPT1C COL1A1 OSCP1 COL4A1 COL6A1 COL9A3 COL12A1 CORT SLCO6A1 ADORA1 TAAR1 ADORA2A ADORA2B KLF14 CPN1 CPN2 GPAT4 MAGEC3 CRHR2 MAGEE2 ADORA3 CRMP1 S100A16 TRIM69 MACROD2 CRY2 CSE1L PIWIL4 CSF2 CSF3 CSK CDAN1 CSTA LYPD4 TICAM1 CILP2 ATP8B3 CREB3L4 HJV PM20D1 CTNND1 PHETA2 CTRL CTSB PLB1 ZNF385B ERFE CTSL XXYLT1 CNTN4 PAQR3 CREBRF SLC2A12 VKORC1L1 KCTD7 CYP2C9 CYP2D7 CYP2D6 CYP3A4 CYP11A1 CYP17A1 CYP21A2 CYP24A1 SLC35G1 CYP27B1 DAPK3 MARCHF10 AKR1C2 TMPRSS6 AAVS1 NLRP6 DIH1 DIO1 DNAH8 DOCK2 DPT DRD5 DUSP5 DUSP9 E2F1 ECT2 EDNRB PGAM5 EIF2S1 DCP1B ARID2 METTL21C MLKL EIF4E EIF4EBP2 ELAVL2 A2M PIKFYVE SPRED2 CRYBG1 TAPT1 ENO2 CCDC171 ERBB4 ERCC2 ERCC4 ESRRB ETFA ETV3 EZH2 F2R F2RL2 F9 F10 F11 FABP3 FANCB MTIF3 FAT2 TMEM26 FCGR1A MARCHF8 FCGR3A PHACTR1 HOXA11-AS THSD7A FDFT1 FDPS FES VASH1 MLXIP ALDOB KLRK1 RAB18 FOXC1 WDTC1 CAND2 KDM4C KDM6B FLNA GPD1L PASK FLNC NBEAL2 FLT3 SIK2 PHLPP1 PSD3 SIK3 DICER1 SIRT5 SIRT4 NCS1 MLYCD TARDBP OTP TRAM1 SEC14L2 DDAH2 FPR2 AMACR MKRN1 LY96 DIANPH ALOX15 PELI3 IL27 FSHB ALPL ANGPTL5 SLC37A4 GABPB1 NPNT TCERG1L GCA SPDEF TAFA5 GALE PRDX5 MOB4 GALR1 POC1A AMBP GALNT2 SGMS1 GEMIN5 EGFL6 SNED1 GORASP2 ZZZ3 ACOT11 IS1 DNM3 CFAP61 KIFBP GATA2 FBXO3 GCH1 NUPR1 OPN1MW GCY GDF2 GDF10 TAS2R12P HCA1 NAP1L5 GFI1 GH2 FETUB GJA3 NPTN GJA8 MLH3 NOX1 FOXP1 INTU NAAA GLS2 HCAR1 SIT1 TMEM97 STK39 MCAT GLI1 DESI1 GLI2 MAT2B GM2AP1 GNA12 GNAQ AMY2A GNRH1 ABO AMY2B GOLGB1 IFNL3 IFNL1 JAKMIP3 H19 OR10A4 UTS2R HID1 CCDC57 CAVIN1 RSPO1 DLL1 FLJ33534 PNPLA1 GPR35 GPR39 FFAR3 GRK4 GRK5 ANK2 GPX4 GRB14 FLVCR1 MCTS1 NOB1 GRIN1 PHPT1 MYLIP BRD7 LGALS13 CXCL1 GRP GSK3A GSK3B GSTA4 MSH6 GTF3A GUCA2A TRPM5 HOOK2 MDFIC GUCY2D NXPH1 HADHA HAS2 HDAC2 HEXA PLA2G2E SOCS7 NRG1 ANXA6 HLA-B HLA-DRB3 HMBS FOXA2 HNRNPA1 HNRNPH1 HOXC9 HOXC10 APC HPR AIRE HES1 BIRC2 HSD17B1 BIRC3 HSPB1 HSP90AA1 APLP2 HTR1D HTR3A HTR6 IARS1 GPIHBP1 ICAM3 ID1 ID3 STING1 SLC35D3 IFI27 MCIDAS IFNGR1 GNAT3 SLC35B2 IGFBP7 CCN1 LCE1A TICAM2 IL1RAP IL2RB IL5 IL9 IL10RA IL16 TNFRSF9 ILK IRF1 AQP9 IRF3 IRF4 IRF7 ABCC6 ITGAD ITGB2 ITGB6 ITPR2 ASPG KCP KCNJ5 KCNJ13 USP17L2 KHK KIR2DL1 KIR2DS1 KIR3DL1 KIR3DL2 CHKB-CPT1B SUMO4 THEMIS KRT31 ACAT2 RND3 LALBA UOX USP17L9P LETM1 LGALS1 LIF LNPEP LOXL2 LRP1 LRP2 LTA LTBP3 LUM CD180 LY75 MIR125A MIR132 MIR136 MIR140 MIR144 MIR145 MIR149 MIR150 MIR17 MIR192 MIR19B1 MIR200A MIR203A MIR204 MIR210 MIR216A MIR22 MIR23B MIR27B MIR28 MIR296 MIR30C1 MIR30C2 MIR30D MIR31 MIR320A NBR1 TNFSF12-TNFSF13 ARRB2 SMAD6 MAOB MARK1 ARSD MARK3 DNAJB3 MBP MC2R ADAM11 MDM2 MEFV MAP3K3 MAP3K5 MGAT3 ASAH1 MIP MLH1 MMP12 MMP15 MMP19 CD200 PLIN5 EEF1A1P11 BECN2 MIR324 MIR326 MIR335 MIR374A MSH2 MSN MST1R MT1B MT2A ATP6 CYTB MTHFD1 ND1 MTR MUC1 MYCL MYH6 MYH7 MYL2 NCAM1 NCK1 ATIC ATM NEFL NFYA NMBR NOTCH2 ATP2A2 NPR1 ATP2A3 NRF1 NTSR1 NR4A2 CISD2 MIR361 MIR377 MIR378A MIR379 MIR383 MIR423 MIR424 OMG P2RX7 P2RY2 P4HB G0S2 NOX3 DELEC1 SERPINB2 PAM CNTN3 NEUROG3 ASAP1 TAS2R14 F11R PDE11A FOXP3 TMED7 PCK2 APH1A PCNA SLC45A2 MZB1 RXFP3 KCNK9 TLR8 RSRC1 PDC PDCD1 DACT1 TAOK3 ATP5F1E PDE4B PRKAG2 DCDC2 SIRT7 LSR PDK1 PDK2 ACSL5 PEG3 ADA2 PEX1 SLC25A3 PHF2 SERPINA4 PIM1 PITX3 PKHD1 PLA2G2A PLA2G5 PLAGL1 PLAU PLCB2 LRP1B PLD1 PLEK PLK1 PLOD2 PLXNA1 PMAIP1 PNOC GPR84 RAB4B CNTN5 PRRX1 ATP7B FAM3B POLE3 NANS TREM2 TREM1 SLC38A2 RETREG1 XRN1 KRT20 RNF216 EXOSC4 ROBO4 POU3F2 DDIT4 EGLN1 POU5F1 ARL15 RBFOX1 PPIA PPIB PPID QPCTL VPS13C CASZ1 CYP2W1 TMEM160 PLAAT2 ULK4 ACP3 MARCHF1 PID1 ATG16L1 MTPAP PRMT6 AVPR1A PPP2R2A MOB1A PSPC1 PPP2R5C AVPR1B LARP6 LAPTM4B IMPACT NPY4R PRB1 MIR31HG MIR429 MIR448 SAGE1 PLXNA3 MIOX BMP2K STAP2 ZNF407 HIF1AN VPS35 CNDP2 SEPTIN11

MBD5 MCTP2 ADCY10 DOK5 CENPJ EAF2 CISD1 PSENEN PRKD1 LMO3 PRKCZ CMAS PRKDC SULF2 MAPK7 MAP2K6 EIF2AK2 ANKH PRNP PROP1 GPCPD1 PARD3 RELN KLK7 SLC2A9 ASAH2 PSEN1 PSG5 SPHK2 CELF4 SEMA3G ARNTL2 PSMB6 DUSP22 RGMA SLC12A9 RPGRIP1 TIGAR LPAR5 CD248 PTCH1 PTGDR PTGDS JPH3 MIR20B AS3MT SLC24A3 PTGS1 MIR496 MIR522 AHRR ZNF608 NLGN2 SEMA6A LRRN1 CHD8 MAGEE1 CPNE5 DENND1A METTL14 PTPN6 MARK4 CCAR2 PTPN11 PTPRB PTPRC PTPRE PTPRJ PTPRS PTBP2 PVALB CXCL16 NLRC4 BCAT1 BCAT2 RASA1 RASA2 TRPV4 ALOXE3 RBMS1 REG1A RGS4 ACTB FAM3A RNASE2 BCL6 EXOC4 ELOVL5 CPAT1 GAS5 TNFRSF17 ROBO1 ROCK1 OPN1SW RPL10 RPL11 RPL29 BCS1L DELYQ11 MIR483 RPS19 RRAS RYR2 S100A4 S100A6 S100A7 S100A12 CFB SAA3P TSPAN31 MSMO1 CCL3 BHMT CCL7 CCL8 CCL14 CCL17 CCL18 CCL22 BID CXCL11 CX3CL1 SDC2 CXCL12 UBE2O DMRTA1 SEL1L NPVF SELENOW MAP2K4 SFPQ SFRP2 POU5F1P3 SRSF1 TRA2B BLVRA MTMR14 SCG5 SH3GL1 POU5F1P4 CLEC7A EBF2 NUCKS1 BMI1 ST6GAL1 ST3GAL2 AIDA RFX7 PPP1R3G NDRG4 NBEAL1 WNK1 SLC2A3 SLC2A5 SLC5A5 RPL7P9 SLC6A12 SLC12A3 SLC16A1 SLC19A1 BMPR2 SLPI SNAI2 SMARCA2 STK33 SMO SMPD1 SNCA SNCG FSCN1 SOAT1 DST SPI1 SPINK1 SPTBN1 SRC ITPRID2 SSTR2 ST2 ST13 ZFP36L1 STAT1 STAU1 STC1 SULT1E1 STXBP3 SULT1A1 SUV39H1 SYT4 TAC1 TAC3 TADA2A TAT TBL1X TBXA2R TCEA1 MIR33B MIR551B MIR593 MIR634 TCN2 BUB1B TFDP1 TFF2 TFF3 TGFB3 TGFBR2 THBS2 TSPO THRA TIE1 TIMP4 TSPAN8 TNNI3 TP73 NR2C2 TRAF6 TRP-TGG3-1 TRPC3 TRPS1 MIR652 TTR MCHR2-AS1 USP17L24 USP17L25 POTEF USP17L26 USP17L27 TUFM USP17L28 USP17L29 USP17L30 OPN1MW2 SERF1B TYRO3 UGT1A UTRN VAV2 VEGFC WAS LAT2 WNT7A WRN YY1 YWHAZ ZFP36 CFAP410 CA5A MIR758 ZBTB16 ZNF214 PRDM2 MOGS FZD5 SEMA3B NABP2 REEP5 ASPSCR1 ELOVL6 APOO MBOAT7 LAP PPP1R3B FN3KRP GHS TBL1XR1 CARF CSPP1 MAP9 RIN3 CALD1 DOCK5 PTGES2 SPSB1 FUZ LHX3 LPAL2 ADAM12 MFAP5 ITIH5 CMIP HMGA2 WNT5B CAMK2A SLC38A1 CAMP GDF5 NCOA3 BAS CAPN1 CAPN2 CAPN3 CAPNS1 NAA10 TKTL1 SERF1A PICALM BRAP ARHGAP24 PARP9 ITCH LOH19CR1 TMTC1 IMMP2L ADGRV1 TAGLN2 HORMAD1 ULK1 CASP8 POLR1B MEHMO MAF1 CHCHD5 NCK2 DOC2B HOPX COL25A1 ZGPAT ZNF469 CNDP1 GPAT3 PERM1 RAX2 MFSD2A SLC35B4 CYP4F2 ABCC11 LGALS12 HELZ2 TSPYL5 MAPKAPK5 BHLHE40 PIAS1 DENR PDXK RUNX1 RUNX1T1 UNC5C PDE5A SLC4A4 STX16 BECN1 TNFRSF25 ADAM19 CBR1 TNFSF12 TNFSF9 TRIM24 FGF18 CES2 NRP1 HCAR3 ASAP2 LDB1 PER3 CCKBR KRIT1 EIF2S2 ACTN3 SNURF WASF1 CCNE1 SIGLEC12 UCN2 SCRN2 MCU TBL1Y PYGO2 USP10 CERS5 PCSK7 ZBED1 LRRFIP1

Supplementary Table S4. 217 overlapping genes between 12 bioactive ingredients of Coptidis Rhizoma and obesity

**Overlapping Genes**
HTR2B BCHE ADRA2B ACHE CYP2D6 CDC42 RPS6KB1 RAC1 AURKB HTR3A<br>IKBKB PPIA PARP2 PIK3CD PIK3CB PIK3CG SRC CHEK1 PTGS2 JAK2 CDK4<br>BCAT2 ICAM1 SELE ALOX5AP MAOB MAPK14 LIMK1 PIM1 PFKFB3 F3<br>HSD17B1 CSF1R MAPK8 TEK TRPM8 PTPN1 FLT3 HSD11B1 MIF OPRM1 OPRD1<br>MAPK1 CTSS CTSL BACE1 CTSK CASP3 DRD2 HTR2A PLA2G2A MDM2 AOC3<br>CFD WNT3A PTGES PREP AVPR1A C5AR1 NAMPT F2R BRS3 OXTR CNR1<br>PER2 FASN P2RX7 CHRM2 AKR1C3 CDK1 PARP1 NR1I2 CYP19A1 TAAR1<br>HSP90AA1 RHOA CLK2 DYRK1B DGAT1 SCD CD38 NR3C2 MAPK10 ROCK1<br>XBP1 ADORA2A ADORA3 SIRT2 AGPAT2 SIRT3 SIRT1 GRK5 FLT1 MAP4K4<br>RPS6KA3 MST1R DRD4 CDK8 FGFR1 STAT3 DRD1 ADRA1A SLC6A3 DRD5<br>ADRB2 ADRB3 HTR2C ADRB1 TBXA2R ABCB1 MAOA MTNR1B PIK3CA ESR1<br>HTR6 ADRA2 AGCK ADORA1 HTR1B PLK1 GSTP1 F2 MAPK9 NAAA ALOX5<br>MAPK3 ILK GSK3A CPT1A ALDH1A1 BRD2 AR PRKCA JAK3 TYK2 NOS2<br>NR3C1 PTGS1 MPO CREBBP PRKDC LIPA HTR1D PDCD4 TLR8 MCHR1<br>CYP1B1 ALDH2 ABCG2 BMP4 CYP1A1 MTOR PRKCB TGFBR2 PDK1 IKBKE<br>TRPV1 TBK1 FGFR3 MARK1 FPR2 NOX4 AKR1B1 XDH IGF1R EGFR GLO1<br>PIK3R1 GSK3B MMP3 CA3 ALOX15 MMP9 MMP2 ALOX12 CA4 NEK2 AKT1<br>PLA2G1B CA5A AKR1C2 AKR1A1 GPR35 MAPT INSR ARG1 PTPRS ESR2<br>SLC22A12 CDK6 TYR AHR ESRRA APP TTR MMP12 TNKS MMP1 BRAF<br>GLI2 ESRRB THRA THRB PRKCZ HPGDS PDE4D ADAM17 GRK2 SLC5A1 TNF<br>AGTR1 ANPEP MMP14 NR1H4 TSPO AKT2 EPHB2

Supplementary Table S5. GO and KEGG pathway data from functional enrichment analysis

Category		Term	Count	Percent (%)	p-value
GO	MF	Protein binding	159	73.27	1.38E-10
		ATP binding	67	30.88	4.33E-20
		Protein kinase activity	38	17.51	4.11E-23
		Protein serine/threonine kinase activity	35	16.13	2.32E-22
		Zinc ion binding	33	15.21	3.63E-05
		Enzyme binding	26	11.98	1.13E-12
		Identical protein binding	26	11.98	1.15E-05
		Kinase activity	23	10.60	5.60E-13
		Protein homodimerization activity	23	10.60	1.76E-04
		Protein kinase binding	17	7.83	2.84E-05
	CC	Plasma membrane	107	49.30	6.84E-18
		Cytosol	88	40.55	2.11E-14
		Cytoplasm	85	39.17	7.18E-04
		Nucleus	81	37.33	1.20E-02
		Integral component of membrane	74	34.10	4.47E-02
		Integral component of plasma membrane	56	25.81	1.54E-15
		Nucleoplasm	55	25.35	1.25E-04
		Extracellular exosome	51	23.50	1.75E-03
		Membrane	45	20.74	3.23E-04
		Mitochondrion	34	15.67	4.25E-05
	BP	Signal transduction	46	21.20	1.79E-11
		Protein phosphorylation	43	19.82	3.49E-24
		Negative regulation of apoptotic process	38	17.51	1.66E-19
		Positive regulation of transcription from RNA polymerase II promoter	37	17.05	1.11E-08
		Response to drug	35	16.13	2.32E-22
		Positive regulation of cell proliferation	32	14.75	5.38E-14
		Inflammatory response	26	11.98	2.14E-11
		Oxidation-reduction process	25	11.52	6.88E-07
		Positive regulation of ERK1 and ERK2 cascade	24	11.06	5.59E-17
		G-protein coupled receptor signaling pathway	24	11.06	1.31E-03
KEGG pathway		Pathways in cancer	48	22.12	3.27E-18
		Neuroactive ligand-receptor interaction	42	19.35	2.74E-19
		Proteoglycans in cancer	33	15.21	3.71E-16
		cAMP signaling pathway	31	14.29	1.79E-14
		PI3K-Akt signaling pathway	31	14.29	2.46E-08
		Rap1 signaling pathway	30	13.82	5.94E-13
		Ras signaling pathway	29	13.36	2.28E-11
		Hepatitis B	25	11.52	1.29E-12
		Focal adhesion	25	11.52	2.47E-09
		FoxO signaling pathway	24	11.06	1.78E-12
		Influenza A	24	11.06	4.41E-10
		Chemokine signaling pathway	24	11.06	1.69E-09
		HIF-1 signaling pathway	23	10.60	1.06E-14
		Sphingolipid signaling pathway	23	10.60	1.40E-12

	Calcium signaling pathway	23	10.60	4.40E-09
	MAPK signaling pathway	23	10.60	2.14E-06
	TNF signaling pathway	22	10.14	1.16E-12
	Serotonergic synapse	22	10.14	2.46E-12
	Hepatitis C	22	10.14	9.10E-11
	Insulin signaling pathway	22	10.14	1.86E-10
	Pancreatic cancer	21	9.68	3.36E-16
	Insulin resistance	21	9.68	1.28E-11
	MicroRNAs in cancer	21	9.68	1.49E-04
	Prolactin signaling pathway	20	9.22	3.58E-14
	ErbB signaling pathway	20	9.22	1.94E-12
	Prostate cancer	20	9.22	2.42E-12
	Toll-like receptor signaling pathway	20	9.22	7.78E-11
	Thyroid hormone signaling pathway	20	9.22	3.38E-10
	Neurotrophin signaling pathway	20	9.22	7.19E-10
	Osteoclast differentiation	20	9.22	3.31E-09
